# Operative Dentistry

**Published:** 1876-11

**Authors:** H. C. Thompson


### OPERATIVE DENTISTRY.

BY DR. H. C. THOMPSON.

*Read before the Maryland State Dental Society.*

Mr. President and Gentlemen:—It is hardly appropriate for a committee on so important a subject as operative dentistry to beg excuse, especially in the very beginning of its report, but when we consider the brief existence of our society and the consequent short time I have had to become acquainted with the members and conversant with their different practices, I feel that there must of necessity occur some void in this report, for which I would beg allowance.

I do not propose to enter into any lengthy discussions relative to the merits or demerits of materials or instruments for filling teeth; that I leave for discussion by the other members of this society; but instead, will remark upon such of them as by their importance demand our greatest attention. I recognize that my theme is broad, and that it is not he who does the most that does it best. Hence, I will endeavor to do justice to a part of this subject rather than injustice to all of it. I do not take too much on myself when I assert that the greatest blessings bestowed upon our craft may be summed up under three heads, viz: Cohesive Foil, Rubber Dam, and Burring Engine. How have they benefitted the dental surgeons? How have they benefitted mankind, who constitute his patients? By bringing about an entire revolution in the practice of operative dentistry, enabling the operator to accomplish results which, had he undertaken a few years since, would have won him the name of fanatic. These improvements enable him to effectually check that indiscriminate sacrifice of the dental organs, making this sacrifice an exception instead of a rule.

Recognizing as we do, how generously these discoveries have been made known to the dental profession, can we do less than bow in homage to their discoverers? The cynic may say that this is nothing more than should be expected from men of natural genius. This is true, to some extent; but how many who possess this genius fail to utilize it because it is too much trouble? Therefore, when these Pioneers of Progress come forward with their valuable contributions, we can but feel that ours is a lasting obligation which time can never cancel. The day has come when it is a matter of impossibility for the dental operator to succeed without embracing the facilities afforded by these valuable improvements. To form an idea of their appreciation, read any dental journal of our country, and almost in every number some worshipper to the Shrine of Perfection contributes his "mite" toward extolling them to the skies.

Where, at one time, the teeth, in many cases, were sacrificed under the forceps, (the then dental surgeon's principal instrument) such teeth are, in the majority of cases, rendered as useful for the purpose of mastication as they ever were. To be sure, this sacrifice was once more necessary, owing to the ignorance of the various methods of treating diseased nerve pulp, and also from the fact of the operator's inability to fill certain cavities of decay (owing to their shape) with gold, the cohesive qualities of which have since been developed. To this last mentioned fact may be attributed the present wide spread prejudice against amalgam and other plastic filling. The operator being unable to place in a gold filling, amalgam was used indiscriminately. But since the discovery of the cohesiveness of gold, there seldom appears a case wherein much satisfaction can not be given our patients, whose interest in the progressiveness of our specialty should be considered equal to our own. Of late much controversy has arisen relative to the best manner of preparing gold previous to inserting it into a cavity of decay. I will not take on myself the responsibility of deciding which is the only proper method, there being so many to decide between, each having its respective advocates, any of whom I have no doubt can make a successful operation after his method. Some claim that in the form of cylinders or pellets the gold can be placed in more rapidly with equal success, while the advocates of ribbons hold that the ribbon is the only form in which gold can be worked into a filling of a proper consistency. Be this as it may, we can but admit that when a skilled operator places in a gold filling, no matter in what form he may have previously prepared his gold, if he exercises his judgment, the filling will save the tooth if the tooth can be saved.

And is it not vain for an advocate of pellets to attempt to convince him who has successfully used ribbons for years, that pellets are the only true form of preparing gold? To be sure the progressive dentist should consider it his duty to give his views to the world, but in doing so he must discriminate between advancing his profession and cherishing a hobby. Only a short time ago the use of heavy foil was considered as something to be avoided. Indeed, so vehement were some that they went out of their way to warn those who had not used it, of the danger of its adoption. Now ask the question in this relation, and what is the answer? That it is considered indispensable in a great many cases, for we find that it gives to a filling (when properly inserted) a surface of much greater density—a surface capable of taking on a better finish, consequently better capable of withstanding the wear of mastication. It is true it is much more difficult to adopt a large body of such gold to the irregular wall of a cavity, but this objection is overcome by using less weighty foil for this purpose. Much discussion has arisen of late from a paper written by a gentleman of some supposed eminence, in which paper he advanced the assertion that it was impossible to make of cohesive gold a filling that would be water tight. Now I would not dispute the gentleman's experience in this relation, but from the experience of those around us who have adopted the use of cohesive foil—by the experience of writers who do not write merely to see their name in print, to admire the comet-like appearance with the D. D. S. forming the tail thereof, but of writers whose communications are for the benefit of their fellow-men, we find this broad statement controverted practically.

But it is generally conceded that greater care is necessary to adapt it to the walls of cavities than where soft foil is used, because every specimen of cohesive gold shows the crystals to be more rigid, consequently less easily conformed to irregular surfaces.

The chief reason for the prejudice borne by a great number of operators against cohesive foil, is that the virtues are spoiled by careless operators, for this latter class recognize that a filling can be pushed into any cavity, and will at least look well and remain in until the deluded patient leaves the office, whereas had soft foil been used the looks would not have been so pleasing to the patient, hence would arise questioning which we all recognize to be the thorn in the side of the charlatan.

The true operator will use cohesive gold only when he wishes to take advantage of its welding properties. This is not always desirable, for when the cavity will admit of it, non-cohesive foil should be used. This is an admitted fact, after years of practical test by the majority of our leading practitioners.

It is very frequently the case that patients will show us fillings that have been in the teeth from fifteen to twenty years, although these fillings are so soft that they have the appearance of never having been condensed, and yet are preserving the teeth. Naturally the question arises—Why is this? It is because being of soft foil they have adapted to the walls of the cavity better than cohesive foil can be adapted, with an equal amount of care; it is because, as a rule, the operations are not as extensive as they are at the present day, and besides the consistency of the teeth was denser than at present. Examine some of our cohesive gold fillings after a few years, and what will be their appearance? You will find them as dense as gold can be made, polished so highly as to make the teeth hold a "mirror up to nature," with often a distinctly blackened frame. With these results before us, will not the majority agree that it is better to utilize the adaptability of the soft foil together with the hardness and welding properties of cohesive foil in order to reach the perfection of gold filling? It is claimed by those who use cohesive exclusively, that in frail teeth harder fillings can be inserted with less danger of fracture of the walls than when soft foil is used. This is an undisputed fact, but it only goes to prove that pressure, when condensing heavy or highly cohesive foil, does not reach the point intended, because the body of gold next the point of the instrument hardens before the pressure reaches that next the walls of the cavity. The result is that when the pressure is renewed in another part of the filling, the part just condensed acts as a lever and draws the edges from the walls of the cavity, consequently the filling will be very hard and would be perfect—were it not just a little too small for the cavity.

This is the reason frail walls stand under so much pressure; but although they withstand the pressure of the instrument, they can not withstand the fluids of the mouth, which insinuate themselves between the enamel and the filling, and the chemical action resulting therefrom.

The operator of to-day, will in many cases bestow double the amount of time on a gold filling that was devoted by the older brother on like cases in his palmy days. There is a cause for this, and it can be stated in a few words. In the present day, the dental operators better appreciate the requirements of the case, and since they adopt the use of cohesive foil, must manipulate it as only such foil will admit of being manipulated, that is, very little at a time; and beside, they bestow more time in the preparation of a cavity of a tooth to receive a filling, for they are taught by experience that the structure of the teeth of each successive generation is less capable of combating the ravages of disease than were those of the generation preceding.

Right here I would dwell on the importance of the thorough removal of fissure! It is true we sometimes see cases where there has been apparently no effort to cut them out, and yet the filling and tooth both in good condition. Such a condition of things is found in the strongest class of teeth only, and is consequently exceptional. But in this day, to leave the very centers of decay around a filling, is nothing less than consigning it to destruction.

We very frequently see cases wherein our judgment is for a time puzzled to determine whether it is better to cut away a fissure that shows no sign of decay, or to excavate and fill only where the disease has made itself apparent. In the majority of such cases we should not hesitate, for each fissure forms a cul de sac, and although decay may not as yet have found the true state of the case, rest assured it will not take long to do so; therefore, when a filling has been inserted into the main cavity only, the gold can never be so placed and finished that there will not be weak barriers against disease. Again, these fissures, forming as they do, cavities of a triangular shape, is it not inconsistent with the laws of mechanics to attempt to condense gold into a triangular cavity with square or round pointed instruments? To be sure it might be suggested that triangular pointed instruments be used, but how can we determine at what angle a plugger must be to meet the requirements of each case. In all cases where the instrument will catch in those fissures, the majority will agree they should be cut out.

I have implied that in the past the removal of these fissures was not as necessary to a perfect operation as it is today. I will modify it and say that it was very necessary, but owing to the difficulty of performing it thoroughly, it was to a great extent neglected, but with the present facilities, their thorough removal is only a matter of a little extra time and labor, of which we should not be chary.

A very able writer once said that "the greatest blessing, when abused, became the greatest curse." This is often verified by the manner of inserting amalgam fillings by some dentists of our cities.

Indeed, a great many do with amalgam as did a great many patriots (?) during the "late unpleasantness"—who sent impecunious cousins into the war just to fill up the ranks; if killed, they were paid for it, while the survivor was praised for his bravery until he really thought himself a patriot. So it is with a great many cheap dentists; not charging sufficient to justify a skillful operation, they place these "poor cousins" —plastic filling materials—into the ranks (of molars) to fill up, and for a time are exalted in the minds of deluded patients, for the reasonable charge for so much work, until the operator considers the work well done. Only lately a case of this kind came under the notice of some of your members; a case where fifteen amalgam fillings were effected in three hours, and yet the operator who accomplished all this received praise worthy of the gods. But we must not condemn this material because of its abuse, for we can but admit that without it or its kind, many a weak molar would have to succumb to the forceps. The great secret of the prejudices against these fillings, is that they are easily inserted, will remain in almost any shaped cavity, and in consequence, serve to hide many a dental abortion. The true operator will embrace any material that in his judgment will save the organ affected.

Relative to rubber dam, too much value can not be placed on its use, and too much praise can not be accorded its generous discover, by the dental profession; its use has become universal, many adopting it to such an extent that they never undertake to place in a filling without first applying the dam to the tooth to be operated on. Its adoption to this extent I can not recommend, because in many cases it subjects our patients to unnecessary suffering when the napkin would answer every purpose. Of course one must exercise judgment in this relation and be governed accordingly. Its application is frequently accompanied by a great amount of trouble to both operator and patients, but where the position and extent of the cavity justifies its use, the benefit derived from it, when properly applied, will compensate for any amount of trouble. In hypersensitive cavities the use of the rubber dam is invaluable, especially where the saliva is of an acid character, for in many cases, by placing the dam over the tooth before excavating, drying the cavity carefully and placing therein a drop of solution of bicarbonate of soda and again drying, the dentine becomes almost devoid of sensitiveness. All who have had experience with the dam will agree with me, that it is not the placing rubber on the tooth that occasions the trouble, but keeping it there when once on. For this latter purpose many appliances have been invented, many of which the majority of this society is familiar with; but let the appliances be perfect, without an abundance of good nature and patience, there will be many cases of failure, for some of them are trying to both; beside we must have the co-operation of the patient, for without the latter, especially if the case be difficult, we had better not undertake the use of the dam. There are many ways of applying it, some of which are attended by considerable pain, let the operator be as delicate as he may in his manipulations. The experience of your committee is that floss silk ligature will answer in the majority of cases, although the metal clamps can be applied without inconveniencing the patient. Lately a manner of placing on the clamps has attracted much attention and seems to meet with general approval; it consists of placing the clamps through the rubber before placing it on the tooth, then inserting the forceps in the clamp, carrying back to the tooth and placing in position, after which the rubber is drawn beneath or above the clamps. This practice often overcomes the necessity of having an assistant to adjust the clamp.

I do not consider it necessary to go into an explanation of the different engines now in use by the profession, as I judge that the majority of the members present are familiar with all. By the use of these instruments it is an easy task to excavate the most difficult cavities, enabling the operator to accomplish results hitherto considered impossible. By means of the different attachments we are enabled to perform operations more thoroughly, with less pain to our patients, and with much less fatigue to the operator. I would call especial attention to the corundum and hard rubber devices. Where there is any amount of separating to be done, these instruments are invaluable, for the steady revolutions of the former while cutting, leave an even surface, after which the rubber disc polishes very highly. The corundum and wood polishing points need only to be used to be appreciated.

In adopting the use of the engines we must not allow ourselves to be led into a too free use of them; for, from the very fact of their abreviating labor, they will in time be in the hands of every empiric of our country who can possibly accumulate funds enough to purchase one. I can venture to say that, in coming years, there will not be an instrument used in dentistry from which more abuse will arise, unless the more enlightened members of the dental profession take precautionary measures to prevent it. The question is, how can it be prevented? By so educating the patient and operator—the latter, that he may be compelled to know what to do; the former, that he may understand what is being done by the operator, and why.

There is one branch of dental practice which, although of great importance, seems to be avoided by writers generally; the reason for this I can not assign. I refer to the operation of extraction. We all agree that it should be considered a "dernier resort" even when the tooth is the seat of severe pain; but since, at times, this operation has to be performed, let it be done properly. By many it is considered a mere operation of strength, and being strong, they attempt the removal of the tooth, notwithstanding the fact of their extreme ignorance of its anatomy, using ill-adapted instruments. The result is that in many cases the crown is crushed or roots fractured, and the patient leaves the office with the consoling information that the roots are, to quote one gentleman's phraseology, "hooked around the jawbone" and can not be extracted. There are many cases where it is impossible for the dentist to avoid fracturing teeth, but to the operator who will study this branch of practice they are rare, for, if he has gained the co-operation of the patient, and shown to him not the visage of a butcher, but of the sympathetic though determined dental surgeon, who has the knowledge which enables him to perform the operation, however disagreeable that may be, as well as the circumstances of the case will allow. But it is frequently the case that the patient can not undergo the pain of extraction. It is under these circumstances that the dentist should advise the use of anæsthetics. No matter how healthy the appearance of the patient, the dentist must consider, before he advise, that, athough there exist what are considered safe anæsthetics, he can not administer any of them without taking on himself an amount of risk just in proportion to the physical condition of the patient, and that one failure would do more injury than a thousand successes will do good. This fact teaches us that we can not devote too much care and study to this important branch of dental surgery to enable us to know when a patient is not a proper subject, physically. When a patient calls for the purpose of inhaling an anæsthetic he places his life in our keeping for the time, and to attempt to administer an anæsthetic without as thorough a knowledge of its effects on each individual temperament, as science and our own powers of perception afford, we do nothing less than commit a crime, which, unfortunately, the law does not reach.

By general consent, nitrous oxide has been accepted as the safest of anæsthetics, from the fact that, although administered to thousands, we have but two or three deaths that could be attributed to the gas, and experience has proven that, when pure and administered by those skilled in its use, it acts more readily, and the effects are more transient, than ether or chloroform. Even though considered safe, we must bear in mind that the condition produced by it is abnormal, and from its power of producing great waste of vital forces, it may frequently produce dangerous symptoms, which should never escape the watchful eye of the operator, who should be always ready to restore the patient to consciousness instantly. Many question the ability of an operator to resuscitate a patient from the influence of nitrous oxide, but practical tests prove that by using a strong current from an electro-magnetic battery a patient can be awakened almost instantaneously. For this purpose the simple Davis & Kidder battery seems to be best adapted, it being easily regulated, simple in construction, and always ready for use. I have seen but one case where it failed to accomplish the desired result. It was that of a young lady who called to have a tooth extracted, the parts being in a highly inflamed condition. Under protest the gas was administered. After taking a few inhalations spasmodic contraction of the epiglottis ensued, the pulse falling to an alarming extent. The tooth was extracted, and the battery applied by means of wet napkins over the poles—the positive under the left axilla and the negative in the right hand. At first a mild current was started, but having no decided effect, it was strengthened to its full capacity, when the patient began to improve rapidly, but on discontinuing the current there came a relapse, each time worse than the preceding. In the meantime wet napkins were placed at the throat, the tongue drawn foward, and spirits of ammonia used freely. After remaining in this condition for more than two hours the patient rallied and was soon able to return to her home. Since then she has felt no ill effects from the gas. But experience of this kind serves to impress on the mind of the dentist the vast responsibility he assumes when he begins the practice of general anæsthesis.

In the administration of any anæsthetic, system in all things should be observed rigidly, for without it we render ourselves liable to have trouble at any time, for which we are not prepared.

When a patient calls on me for the purpose of inhaling an anæsthetic, after examining the case, I prepare my operating chair which is as follows: The battery is placed behind the chair, with poles over the arms thereof, convenient to myself and assistant; the forceps to be used are placed on the bracket in front of the chair and covered with a napkin; spirits of ammonia convenient to my reach, cold water and the inhaler resting on the bracket ready for use. After this preparation the patient is invited to the chair. I studiously avoid letting the patient see this preparation, because if he be at all nervous his mind is proportionately active and this would only increase the activity, my desire being to have the mind as quiet as possible under the circumstances. If possible to prevent it; while the patient is losing consciousness, no unnecessary sound should be heard about the office, but instead, the subdued but steady voice of the operator enjoining the patient to breathe freely and keep the eyes closed. Instead of this how often do we hear of ignorant bystanders or officious friends making frivolous suggestions while the patient is losing consciousness—ideas which often run into violent actions of screaming and struggling, which is often attributed to the anæsthetic by skeptics.

There is one point in the administration of nitrous oxide to which I would call attention. It is that of allowing the patient to remain under its influence for any length of time after the operation is finished. This I always avoid, because I consider it the most critical stage—the stage in which, if there is going to be a dangerous reaction, it usually occurs, for now all local irritation is removed and it depends on nature entirely; whereas if steps be taken to revive the patient immediately, we have little cause to fear bad results.

There are circumstances under which an operator should not undertake the administration of nitrous oxide—certain disorders whose character precludes its successful use, and the dentist should be capable of diagnosing; in functional or organic diseases of the heart, active congestion or inflammation of the lungs or kidneys, or any derangement of the brain or spinal cord, or when there exists a general plethoric condition of the system.

The real danger consists in its adding a foreign element to the blood which inteferes with its proper function, and until the dentist understands the effects of nitrous oxide, where there is a tendency toward any of these disorders, it is better that he should not administer it, for the excessive stimulating effect, apparent from the lividity present in certain stages, may develop diseases which have remained latent for years, and only required some such violent action to bring them to light.—*Proceedings of Md. State Dental Society.*

